# Health Information–Seeking Patterns of the General Public and Indications for Disease Surveillance: Register-Based Study Using Lyme Disease

**DOI:** 10.2196/publichealth.8306

**Published:** 2017-11-06

**Authors:** Samuli Pesälä, Mikko J Virtanen, Jussi Sane, Pekka Mustonen, Minna Kaila, Otto Helve

**Affiliations:** ^1^ University of Helsinki Helsinki Finland; ^2^ National Institute for Health and Welfare Helsinki Finland; ^3^ Duodecim Medical Publications Ltd Helsinki Finland; ^4^ Public Health Medicine, University of Helsinki and Helsinki University Hospital Helsinki Finland; ^5^ Pediatric Research Center, Children’s Hospital, University of Helsinki and Helsinki University Hospital Helsinki Finland

**Keywords:** search engine, evidence-based medicine, medical informatics, information systems, communications media, Lyme disease, infodemiology, infoveillance, surveillance

## Abstract

**Background:**

People using the Internet to find information on health issues, such as specific diseases, usually start their search from a general search engine, for example, Google. Internet searches such as these may yield results and data of questionable quality and reliability. Health Library is a free-of-charge medical portal on the Internet providing medical information for the general public. Physician’s Databases, an Internet evidence-based medicine source, provides medical information for health care professionals (HCPs) to support their clinical practice. Both databases are available throughout Finland, but the latter is used only by health professionals and pharmacies. Little is known about how the general public seeks medical information from medical sources on the Internet, how this behavior differs from HCPs’ queries, and what causes possible differences in behavior.

**Objective:**

The aim of our study was to evaluate how the general public’s and HCPs’ information-seeking trends from Internet medical databases differ seasonally and temporally. In addition, we aimed to evaluate whether the general public’s information-seeking trends could be utilized for disease surveillance and whether media coverage could affect these seeking trends.

**Methods:**

Lyme disease, serving as a well-defined disease model with distinct seasonal variation, was chosen as a case study. Two Internet medical databases, Health Library and Physician’s Databases, were used. We compared the general public’s article openings on Lyme disease from Health Library to HCPs’ article openings on Lyme disease from Physician’s Databases seasonally across Finland from 2011 to 2015. Additionally, media publications related to Lyme disease were searched from the largest and most popular media websites in Finland.

**Results:**

Both databases, Health Library and Physician’s Databases, show visually similar patterns in temporal variations of article openings on Lyme disease in Finland from 2011 to 2015. However, Health Library openings show not only an increasing trend over time but also greater fluctuations, especially during peak opening seasons. Outside these seasons, publications in the media coincide with Health Library article openings only occasionally.

**Conclusions:**

Lyme disease–related information-seeking behaviors between the general public and HCPs from Internet medical portals share similar temporal variations, which is consistent with the trend seen in epidemiological data. Therefore, the general public’s article openings could be used as a supplementary source of information for disease surveillance. The fluctuations in article openings appeared stronger among the general public, thus, suggesting that different factors such as media coverage, affect the information-seeking behaviors of the public versus professionals. However, media coverage may also have an influence on HCPs. Not every publication was associated with an increase in openings, but the higher the media coverage by some publications, the higher the general public’s access to Health Library.

## Introduction

### Background

Internet users seeking health information on the Web begin their search using general search engines such as Google, Bing, or Yahoo [1-5]. Some may start searching on a social networking site such as Facebook [1]. Adults looking for health information from the Internet and social media, sometimes called *online diagnosers,* are more likely to be women, younger adults, and people who have higher education and better household earnings [1,3,6]. Users explore only the first few links from the general search engine results [7], and when assessing the reliability of the website, they primarily look for the source, professional design, and signs of scientific or official touch [7]. General search engines, however, cannot profile their users [8], although the characteristics and behavior of those seeking health information have been assessed [1]. Those seeking health information from the Internet consist of both the general public and health care professionals (HCPs) [4,9]. Health-related information-seeking behavior may be affected by several factors such as personal health disorders, thirst for knowledge, or even media coverage [1,10]. Information is transmitted to large audiences using both conventional media (eg, television, newspapers, and radio) and digital media (ie, Internet and mobile). The Internet has allowed information to spread fast and far. In 2013, a random-sampled survey (4750 people in the age group of 16 to 89 years) from population information system in Finland was carried out via telephone interview. It showed that 86% (4085/4750) of Internet users read media websites, 76% (3610/4750) searched information from Wikipedia or similar wikis, and 56% (2660/4750) participated in social networks such as Facebook or Twitter [11]. In 2013, 80% (3800/4750) of all households in Finland had broadband Internet [11]. Of health information topics sought after on the Web, specific diseases are the most common [1], including disease with a seasonal incidence.

Lyme disease is a tick-borne bacterial infectious disease caused by a spirochete (*Borrelia burgdorferi sensu lato*) [12], which usually occurs in northern temperate climate zones worldwide [13], including Northern Europe (Nordic countries), Central and Eastern Europe, and North America (the United States and Canada). Incidences of Lyme disease differ depending on the month of the year (seasonal variation) and its specific ecological conditions, thus, making the risk of Lyme disease infection greatest between late spring and autumn [12]. In Finland, located in Northern Europe, the incidence of Lyme disease has increased, and seasonal and regional variation is apparent [13-16]. A bite from an infected tick and the resulting human infection typically occur when people spend time outdoors. Due to seasonal tick activity, the clinical manifestations of Lyme disease [12,13,17] mostly appear during summer [13,17]. In the early stage of the disease, local reddish ring-form skin rash, so called erythema migrans, occurs 3 to 30 days after exposure. Systematic symptoms such as fever, headache, muscular pain, and tiredness are common. Patients diagnosed early with localized Lyme disease can be treated with an oral antibiotic therapy [13,17]. If left untreated, the infection may disseminate throughout the body via bloodstream which results in, for example, joint inflammation and neurological and cardiac manifestations. The most common Internet queries on health information focus on specific diseases and medical treatments [1], including Lyme disease.

When searching current health issues, the searches used to access databases where this information is located may be utilized for disease surveillance. However, early warning systems may not monitor information only on a true epidemic but also on an epidemic of fear (*fear epidemiology*) [10,18]. The use of population health technologies during the severe acute respiratory syndrome (SARS) epidemic demonstrated that an epidemic of fear may trigger changes in collective searching behavior gathered by early warning systems. Thus, false positive warnings may lead to media reports and then affect the public’s search behavior on the Internet [18]. Notably, the general population’s awareness of currently common infectious diseases may have an effect on health information searches on Google, which could be escalated by the media [8,10]. Along with current common infectious diseases, Lyme disease has also stirred interest among Internet users who have searched information related to Lyme disease via Google [5]. These search data from Google Trends have approximated the trends in seasonality and spatial distribution previously identified in Lyme disease [5]. As in the SARS epidemic [18], Lyme disease could also trigger an epidemic of fear, especially at the time of extensive media coverage on Lyme disease. This may lead to a positive feedback loop between Lyme disease searches and media coverage [18]. Two terms are defined when framing methods on health-related Internet information and epidemiological data [19]: infodemiology (information epidemiology) and infoveillance (information surveillance). Infodemiology is defined as a discipline within public health informatics that studies information in an electronic medium or in a population, with the aim of informing public health and public policy. When infodemiology data are used for surveillance purposes, the term used is infoveillance [19]. Lyme disease, serving as a well-defined disease entity, was used in our previous study [14] where we showed that HCPs’ searches of Internet evidence-based medicine sources coincided with national register-based data on the geographical findings of Lyme disease diagnoses. Lyme disease searches of Internet medical databases presented seasonal and regional variation, and a suggestion was made to consider searches as an additional information source for disease surveillance. Although Internet users’ health information–seeking behavior has been studied previously [1-3,7,8], little data exist on the general public’s health information searches of a dedicated medical database on the Internet. Therefore, Lyme disease was chosen as the indicator for our study to be evaluated from Internet medical portals.

Duodecim Medical Publications Ltd (owned by the Finnish Medical Society Duodecim) publishes a wide selection of medical information targeted at HCPs [20]. It also produces and maintains an Internet medical portal called Health Library (Terveyskirjasto in Finnish) aimed at the general public (population of Finland: 5.5 million people, 2016 [21]). This publicly available portal consists of more than 10,000 medical articles that were opened over 50 million times in the year 2016. Each opened article is tracked in a log file. The articles in Health Library follow the guidelines published on the Internet-based commercial portal service (Terveysportti), where the principal service is the Physician’s Databases, produced and maintained by Duodecim Medical Publications Ltd. Quality criteria of Health on The Net [22] are met in the production process of the articles in Health Library. Physician’s Databases includes point-of-care Evidence-Based Medicine Guidelines designed for clinical practice comprising 1300 primary care practice guidelines. In the guidelines, more than 4000 treatment, medication, or diagnostic recommendations are linked to quality-graded evidence summaries and further to Cochrane full-text reviews when available [23]. When producing the guidelines, Duodecim Medical Publications Ltd follows the process accredited by the National Institute for Health and Care Excellence (NICE). The databases also include, for example, 120 National Current Care Guidelines published by Duodecim Medical Society, access to the Cochrane library, *Duodecim Medical Journal*, *Finnish Medical Journal*, acute care database, drug databases, search engine for ICD-10, and procedure codes. Every year, approximately 15 million health-related articles are opened from Physician’s Databases. Over 500 medical professionals within their own field of expertise participate in updating and developing the articles. Physician’s Databases is available to HCPs throughout the Finnish health care system by employers; thus, the health care centers and hospitals purchase the right to use the service. The users of databases can be tracked in primary and specialized health care and also in pharmacies by an Internet protocol address included in a log file. Approximately two-thirds of Physician’s Databases’ users consist of physicians working in Finland (personal communication with P Mustonen, August 21, 2017), where there were over 20,000 working-age physicians in 2016 comprising 60% (12,507/20,970) females and 40% (8463/20,970) males [24]. Other users include nurses and pharmacists. The contents of both Health Library and Physician’s Databases are in Finnish.

### Hypotheses

The primary aim of our study was to compare the general public’s openings of Health Library articles on Lyme disease to HCPs’ openings of Physician’s Databases articles on Lyme disease seasonally throughout Finland from 2011 to 2015 and to evaluate how information-seeking trends from Internet medical databases differ seasonally over a 5-year period (January 2011-December 2015). We hypothesized that the timing of the general public’s and HCPs’ article opening on Lyme disease is mainly similar, thus, making it possible to use the general public’s article openings as an additional source of information for disease surveillance. However, we also assumed a priori that patterns would contain some differences in start and end points between openings in the Health Library and Physician’s Databases. The secondary aim of our study was to evaluate whether media publications on Lyme disease are associated with the general public’s Health Library openings outside epidemic seasons. The hypothesis was that media coverage has an influence on the general public’s article opening related to Lyme disease.

## Methods

### Study Design and Data Collection From Internet Medical Portals

We carried out a descriptive register-based study on the general public’s and HCPs’ article openings related to Lyme disease from Internet medical portals to compare logs to evaluate seasonal variations of Lyme disease across Finland from 2011 to 2015. We retrieved retrospectively logs of both articles in the Health Library and Physician’s Databases by assessing the number of openings of Lyme disease articles weekly by the general public and HCPs, respectively. The Health Library logs include only data on the article openings of Lyme disease across the entire country with no geographically distributional data, whereas the Physician’s Databases logs include both openings and searches on Lyme disease for all 21 health care districts in Finland. Therefore, the article openings of Lyme disease for the whole country were chosen to allow comparable data from Health Library and Physician’s Databases. Users may access the Health Library database through different paths. Less than a fifth of users are not directed to Health Library via Google (personal communication with P Mustonen, May 4, 2017). This seems to be the case in Lyme disease, as most search Lyme disease from a general search engine such as Google by using words *borrelioosi* (borreliosis in Finnish) or *borrelia.* Health Library’s article on Lyme disease is one of the first links to come up directing the information seeker to the Health Library database, whereas others use the Health Library’s home page link from the Web browser’s address bar to link directly to the Health Library database. Along with accessing Internet medical databases, users may also browse media websites, including information on Lyme disease.

**Table 1:** The top five Finnish media websites, their types, and the number of weekly browsers.

Media website	Type of media	Number of weekly browsers on media website in December 2013 (week 50)
Helsingin Sanomat	The largest daily subscription newspaper	1.6 million
Ilta-Sanomat	Tabloid	2.6 million
Iltalehti	Tabloid	2.8 million
MTV	The commercial television station	1.6 million
Yle	The national public broadcasting company	1.8 million^a^

^a^In 2015 (week 50).

### Collection of Publications From Media Websites

The three largest and most influential nationwide media companies in Finland are Sanoma, Yleisradio (Yle), and Alma Media [11]. Sanoma consists of the largest national subscription daily newspaper (Helsingin Sanomat) and a tabloid (Ilta-Sanomat). Alma Media consists of a commercial television station (MTV) and a tabloid (Iltalehti). Yle is the national public broadcasting company in Finland. In addition to printed (the daily newspaper, Helsingin Sanomat, and tabloids Ilta-Sanomat and Iltalehti) or broadcasted (television stations, MTV and Yle) information, these media also provide information to consumers via digital platforms, for example, on their websites. We chose these five media for further study because of the large number of weekly browsers on their websites in 2013 [11,25]. In December 2013, the number of website browsers ranged from 1.6 to 2.8 million per week. The data on Yle website browsers was 1.8 million per week in January 2015, whereas the data were not available for 2013. In 2013, a random-sampled survey via telephone interview (4750 people included) showed that Internet daily reached 84% (3990/4750) of Finnish people aged 15 to 69 years, 83% (3943/4750) of females and 85% (4038/4750) of males [11]. A total of 88% (4180/4750) and 77% (3658/4750) daily reached the Internet mass media in the younger age group of 15 to 44 years and older age group of 60 to 69 years, respectively [11]. The top five Finnish media websites with their characteristics are shown in Table 1. Each media website has a search functionality on their home page allowing consumers to search for the information they desire. We collected publications on Lyme disease by searching the words *borrelioosi* and *punkki* (borreliosis and tick in Finnish) using the websites’ search functionality. Articles on Lyme disease were categorized by publication date for every week to be comparable to weekly openings in the Health Library and Physician’s Databases.

## Results

### Visually Similar Patterns

**Figure 1:**
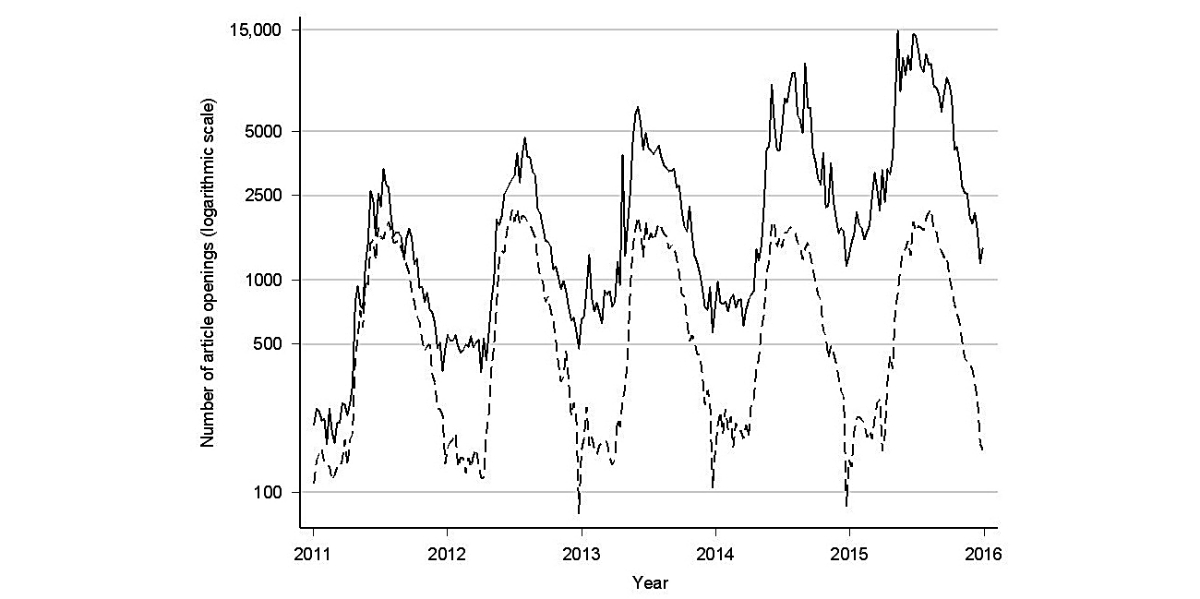
The general public’s article openings on Lyme disease in the Health Library (solid line) and health care professionals’ (HCPs’) article openings on Lyme disease in the Physician’s Databases (dashed line) across Finland from 2011 to 2015.

Our study showed visually similar seasonal patterns in the general public’s and HCPs’ article openings on Lyme disease. The seasonal variation across Finland from 2011 to 2015 is shown in Figure 1. The general public’s article openings related to Lyme disease start at the beginning of May, peak from May to September, and then decline to the lowest point from December to April. HCPs’ article openings on Lyme disease start rapidly at the end of April, peak from June to August, and then decline to the lowest point from December to January. The openings of Health Library and Physician’s Databases peaked at 14,956 in May 2015 and at 2144 in July 2012, respectively. The openings of Health Library were lowest at 169 in February 2011 and Physician’s Databases at 79 in December 2012. From 2011 to 2015, the general public’s article openings on Lyme disease considerably increased in both the maximum (from 3329 to 14,956, 4.5-fold increase) and minimum values (from 169 to 1197, 7.0-fold increase), whereas HCPs’ maximum article openings per week mostly remained constant (from 1868 to 2132, 1.1-fold increase). The number of maximum and minimum article openings by year in the Health Library and Physician’s Databases are shown in Table 2.

### Three Off-Season Peaks

**Figure 2:**
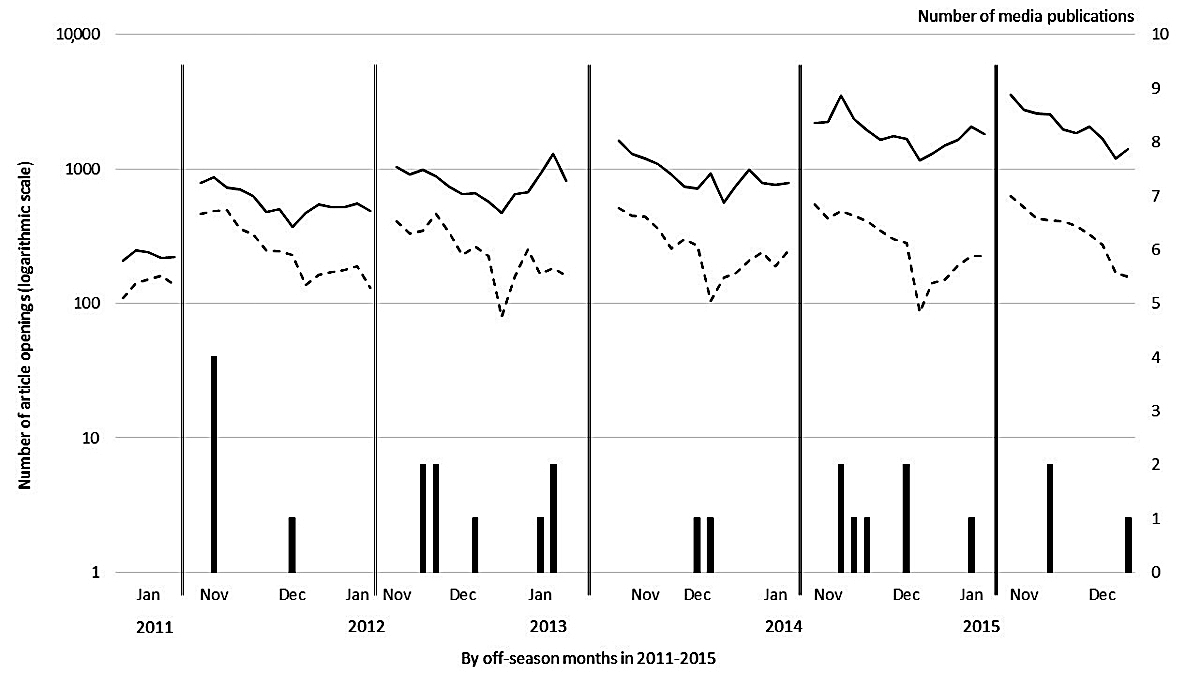
The general public’s article openings on Lyme disease in the Health Library (solid line) and health care professionals’ (HCPs’) article openings on Lyme disease in the Physician’s Databases (dashed line) across Finland during Lyme disease off-season months from 2011 to 2015. Vertical bars stand for media publications.

The number of media publications on Lyme disease published outside epidemic seasons from 2011 to 2015 are shown in Figure 2. From 2013 to 2014, there were three off-season peaks in openings occurring simultaneously with media publications. In January 2013, three media publications occurred simultaneously as a peak in the general public’s article openings, whereas a peak in the HCPs’ article openings appeared before the publications. In December 2013, peaks in HCPs and nonprofessionals’ article openings were seen simultaneously with two media publications. In November 2014, two publications were simultaneously present with article openings in the Health Library.

### Publications From Media Websites

Table 3 shows the number of Lyme disease media publications released on the top five Finnish media websites during off-season months from 2011 to 2015. A total of 25 media publications were retrieved from media website platforms comprising 21 text articles, two text articles with a notice of TV documentary, one notice of TV documentary, and one radio program. Publications were divided into three categories: institutional articles, personal stories, and other publications on Lyme disease. The 15 institutional articles included university or research institution publications or a specialist’s view. The 7 personal stories included a person’s experience on Lyme disease. One publication included both the institutional view and personal story. Two other articles included journalists’ reports on ticks or Lyme disease excluding institutional or personal views. The data are shown in Table 3 and in Multimedia Appendices 1-3.

**Table 2:** The general public’s article openings on Lyme disease in the Health Library and health care professionals’ (HCPs’) article openings on Lyme disease in the Physician’s Databases across Finland from 2011 to 2015. The annual number of the maximum or minimum article opening shows when the opening took place (month, week) during each year.

Article openings on Lyme disease		Year				
		2011	2012	2013	2014	2015
**Health Library**						
	Maximum number of article openings per year	3329	4660	6505	10430	14956
	Maximum opening month per year	July	July	June	September	May
	Maximum opening week per year	28	31	23	36	20
	Minimum number of article openings per year	169	368	566	608	1197
	Minimum opening month per year	February	April	December	March	December
	Minimum opening week per year	6	14	52	12	52
**Physician’s Databases**						
	Maximum number of article openings per year	1868	2144	1977	1874	2132
	Maximum opening month per year	July	July	June	June	August
	Maximum opening week per year	30	29	23	23	33
	Minimum number of article openings per year	110	79	105	86	132
	Minimum opening month per year	January	December	December	December	January
	Minimum opening week per year	1	52	52	52	2

**Table 3:** The number of Lyme disease media publications released on the top five Finnish media websites during off-season months (January, November, and December) from 2011 to 2015. Publications are divided into institutional articles (university or research institution or specialist’s view), personal stories, or other articles. The type of data retrieved from website’s platform is placed in the parentheses.

Year	Month	Week	Number of media publications per month	Media (website)				
				Helsingin Sanomat	Ilta-Sanomat	Iltalehti	MTV	Yle
2011	January		-					
2011	November	45	4	Institutional (text)		Institutional (text)		Institutional (radio program), other^a^ (text)
2011	December	51	1					Institutional (text)
2012	January		-					
2012	November	46-47	4	Institutional (2 texts)	Personal story (text)			Institutional (text)
2012	December	50	1		Personal story (text)			
2013	January	3-4	3		Institutional (text)		Institutional (text)	Personal story and institutional (text)
2013	November		-					
2013	December	50-51	2		Personal story (text)	Institutional (text)		
2014	January		-					
2014	November	46-48	4	Personal story (text including notice of TV documentary on Yle), other^b^ (text)	Personal story (notice of TV documentary on Yle)			Institutional (text including notice of TV documentary on Yle)
2014	December	51	2					Institutional (2 texts)
2015	January	4	1		Personal story (text)			
2015	November	47	2	Institutional (text)				Institutional (text)
2015	December	53	1	Personal story (text)				

^a^A journalist reports ticks and Lyme disease.

^b^A journalist reports the different kind of ticks.

^c^A hyphen (-) and blank cell indicate that no publications are available.

## Discussion

### Principal Findings

To our knowledge, this is the first study on the general public’s information-seeking behavior on Lyme disease from a dedicated Internet medical database. We found the information-seeking behavior of the general public and HCPs on Lyme disease to share a visually similar temporal pattern (Figure 1), which resembles the trend demonstrated by epidemiological data [13,14,16]. In addition, the general public’s opening patterns from Health Library appeared more seasonally fluctuating, and they increased over time in comparison with the rather stable HCP-opening patterns of Physician’s Databases. In addition, we found occasional associations between Health Library article openings and media publications on Lyme disease during off-season months.

Visually similar temporal patterns in information-searching behaviors between the general public and HCPs mirror the trend seen in epidemiological data on Lyme disease. Due to known seasonal and regional variation of Lyme disease and the conclusions in our previous study [14] using HCPs’ Internet searches on medical databases as a supplementary source of information for disease surveillance, we suggest that the general public’s article openings from Internet medical databases should be considered as an additional information source for disease surveillance. Such a conclusion should be drawn very carefully, however, and further studies are needed.

From 2011 to 2015, the general public’s article openings from Health Library appeared to fluctuate more and showed a net increase compared with the seasonal steadiness and absence in weekly fluctuation in openings from Physician’s Databases performed by HCPs. In Finland, the incidence of Lyme disease has increased and also geographically expanded [16]. Therefore, we speculate that across the country, the general public is more aware and is seeking more information on the disease. In addition, the significant increase in the article openings of Lyme disease in the Health Library may be the result of an increase in net openings in the Health Library. In addition, it is possible that whereas HCPs have easier access to English-language websites, a language barrier with the general public could direct more traffic to Health Library, which consists of articles in Finnish. The increasing minimums in openings from Health Library (7.0-fold increase) compared with steady openings from Physician’s Databases are clearly present, which indicates the general public’s interest in Lyme disease also in the wintertime. It is also likely that HCPs are better informed on Lyme disease than the general public, thus, showing less fluctuation in opening patterns. The general public has shown a seasonally different interest on Lyme disease compared with HCPs. Publications on Lyme disease released in the media may be one of the factors affecting the public’s different information-seeking behavior.

Most media publications were text articles (21/25) and institutional texts (15/25). Considering the total of published articles, no clear relation between published institutional texts or personal stories and the general public’s information-searching was found (Multimedia Appendices 1 and 2). However, if merging all types of publications (personal stories, institutional, and other publications), three opening peaks were associated with publications. The following peaks in searches by the general public coincided with released media publications. In January 2013, there were two institutional texts and one personal story with institutional view. In December 2013, there was one institutional text and one personal story. In November 2014, there were two personal stories with a notice of TV documentary on Lyme disease. It is possible that not only a certain type of publication changes searching behavior among the general public but actually a variety of them do, especially when published in a short period of time, ranging from a day to 2 weeks. In fact, a peak occurred with the public’s openings in November 2014, perhaps caused by two personal stories with notices of TV documentaries on Lyme disease, thus, suggesting that personal stories trigger the public to watch the Lyme disease documentary on TV and then start searching for further information from Health Library. We hypothesized that the multiple-peaked patterns caused by vigorously fluctuating openings in the Health Library during both maximum and minimum seasons could have been affected by media publications. However, the general public’s article openings outside epidemic seasons are not consistently caused by publications, although three peaks could be associated with media coverage.

### Comparison With Prior Work

Google search engine log data on current diseases among the general population, for example, people’s queries on influenza or acute respiratory or flu-like symptoms, have been used for disease surveillance [8,10,19]. Infodemiology studies health information on the Internet, such as log data on influenza, to improve public health [19]. Monitoring influenza, infoveillance has been used for assessing the epidemiology of influenza but with conflicting results. It included substantial flaws in geographic scales and timing, such as overestimating the intensity of the epidemic and missing the first wave of the influenza pandemic [8]. In addition, general search engines could not characterize their users, which consisted of both HCPs and the general public. We have previously demonstrated that Lyme disease searches by HCPs in evidence-based medicine databases on the Internet aimed at HCPs coincided with diagnoses, suggesting these searches could be used as an additional information source for disease surveillance [14]. However, it should be noted that not only do the diseases among the general public affect their information-seeking on the Internet but also health-related publications in the media and a fear of disease epidemic may have an influence on Internet searching behavior [8,10,19], also in the case of Lyme disease [12,26].

### Limitations

The study includes certain limitations. Those searching information on Lyme disease using Google are presented with a large number of potential informational websites. Those choosing to proceed to the Health Library website may be more health conscious or more capable of filtering medical data than people not familiar with this. Although the regional variation of Lyme disease incidence in Finland is known [15,16], the geographical diversity data on Health Library openings are not available, and therefore, geographical comparisons could not be made. In addition, possibly because of the small number of off-season publications, the association between every media publication and Health Library opening during Lyme disease off-season months could not be defined, even if some publications did occur at a peak in Health Library or Physician’s Databases article openings. It is, however, worth noting that not only the general public but also HCPs may have been influenced by media publications. Although we collected publications from media websites with the largest number of page browsers, Lyme disease publications on less frequented websites may also exist. We cannot rule out that some visitors in Health Library could have been HCPs. Furthermore, visitors other than HCPs could have accessed the Physician’s Databases as well. However, we consider the strengths of our study to be its timeliness (real-time Internet databases) and representativeness (HCPs in the case of Physician’s Databases).

### Conclusions

We found that the general public’s searching behavior on Lyme disease from medical Internet databases has considerably increased during summertime and wintertime from 2011 to 2015. This indicates that as interest in Lyme disease coincides with increasing numbers of Lyme cases, the Internet openings could be used as a supplementary source of information for disease surveillance. Vigorously fluctuating seeking behaviors outside epidemic seasons was associated with media coverage on Lyme disease only occasionally. Not every publication was associated with an increase in openings but the higher the media coverage by some publications, the higher the general public’s access to Health Library. Further research will be needed to validate our method and apply it to other current diseases published in the media.

## Appendix

Multimedia Appendix 1 The general public’s article openings on Lyme disease in the Health Library (solid line) and HCPs’ article openings on Lyme disease in the Physician’s Databases (dashed line) across Finland during Lyme disease off-season months from 2011 to 2015. Vertical bars stand for Lyme disease media articles published by universities or research institutions or a specialist’s view (institutional texts).

Multimedia Appendix 2 The general public’s article openings on Lyme disease in the Health Library (solid line) and HCPs’ article openings on Lyme disease in the Physician’s Databases (dashed line) across Finland during Lyme disease off-season months from 2011 to 2015. Vertical bars stand for media publications on a person with Lyme disease (personal stories).

Multimedia Appendix 3 The general public’s article openings on Lyme disease in the Health Library (solid line) and HCPs’ article openings on Lyme disease in the Physician’s Databases (dashed line) across Finland during Lyme disease off-season months from 2011 to 2015. Vertical bars stand for media publications other than personal stories or institutional publications in the media.

## Abbreviations

HCPs: health care professionals
NICE: National Institute for Health and Care Excellence
SARS: severe acute respiratory syndrome
