# Supplementary figures and images for: Health Information–Seeking Patterns of the General Public and Indications for Disease Surveillance: Register-Based Study Using Lyme Disease
Source: JMIR Public Health Surveill. 2017 Nov 6;3(4):e86. doi: 10.2196/publichealth.8306 (PMC5696583; doi:10.2196/publichealth.8306)

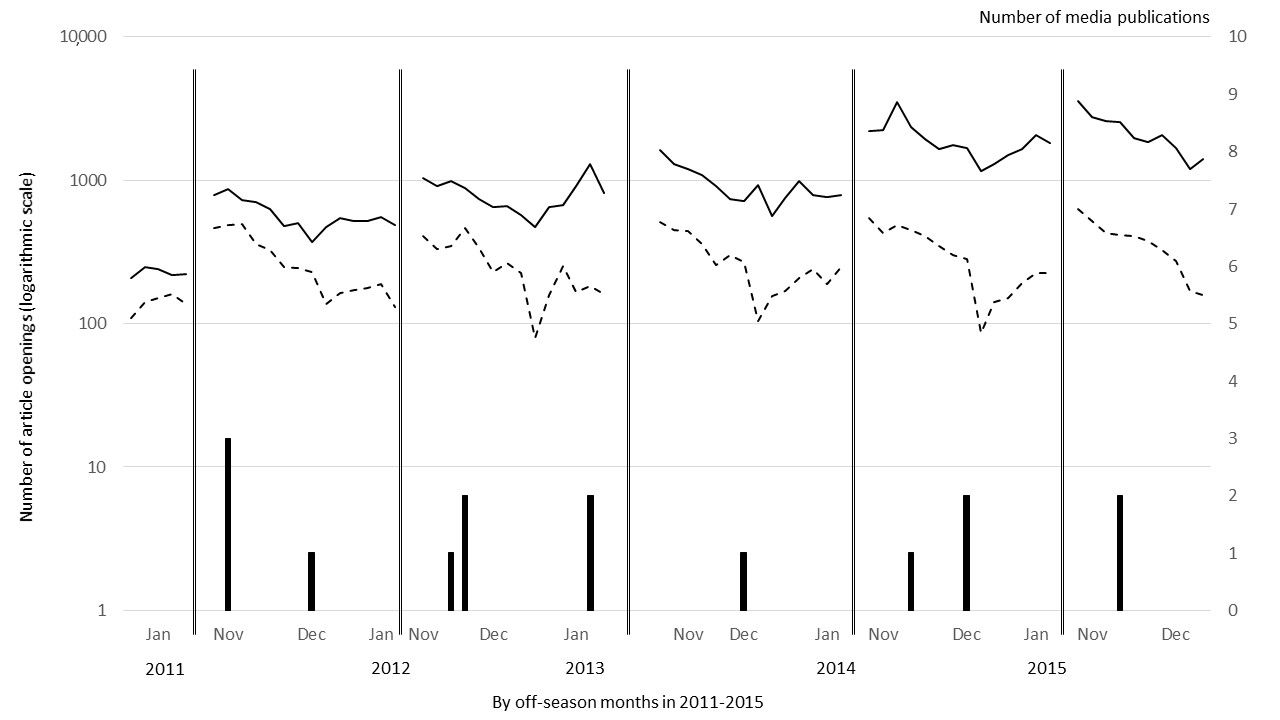

Supplement: Multimedia Appendix 1 [file publichealth_v3i4e86_app1.jpg]

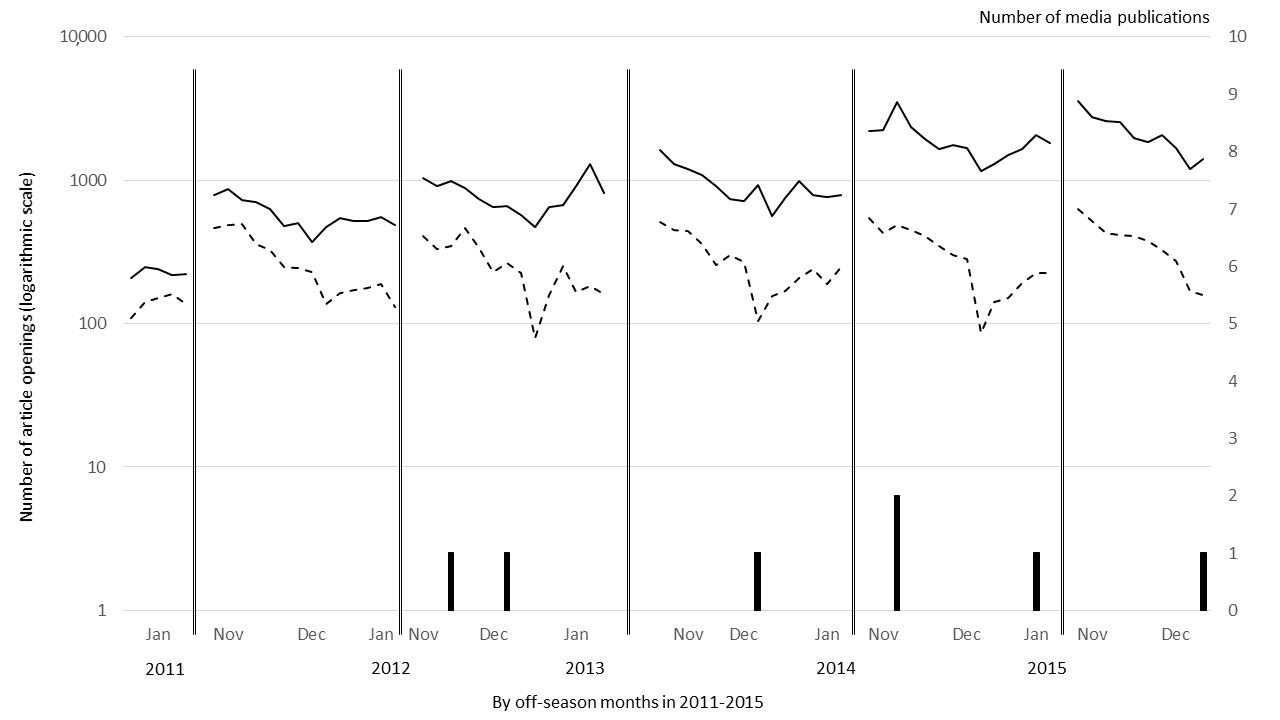

Supplement: Multimedia Appendix 2 [file publichealth_v3i4e86_app2.jpg]

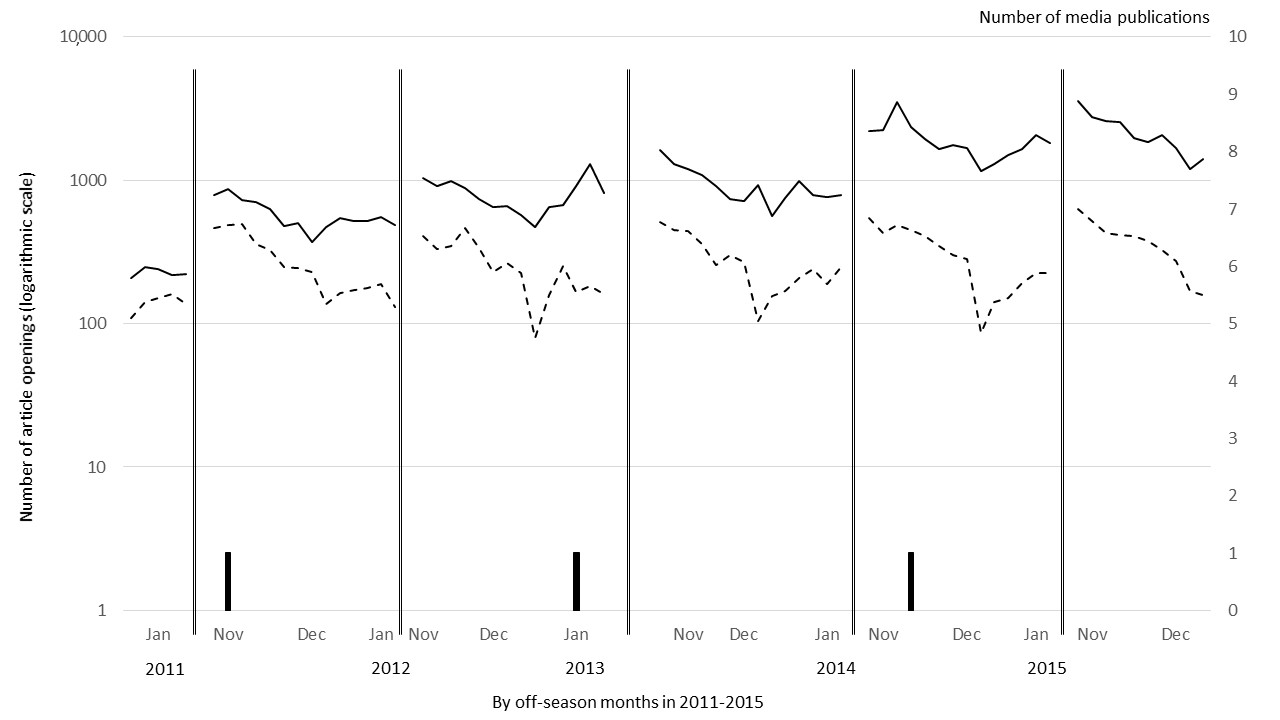

Supplement: Multimedia Appendix 3 [file publichealth_v3i4e86_app3.jpg]
